# Shear Wave Elastography for Assessing Liver Stiffness in HCV-Infected Kidney Transplant Recipients after Direct-Acting Antiviral Treatment: A Comparative Study with Magnetic Resonance Elastography

**DOI:** 10.3390/jcm12247547

**Published:** 2023-12-07

**Authors:** Salma Almutawakel, Fabian Halleck, Michael Dürr, Ulrike Grittner, Eva Schrezenmeier, Klemens Budde, Christian E. Althoff, Bernd Hamm, Ingolf Sack, Thomas Fischer, Stephan R. Marticorena Garcia

**Affiliations:** 1 Department of Radiology, Charité—Universitätsmedizin Berlin, Corporate Member of Freie Universität Berlin, Humboldt-Universität zu Berlin, and Berlin Institute of Health, Charitéplatz 1, 10117 Berlin, Germany; salma.almutawakel@charite.de (S.A.);; 2 Department of Nephrology and Medical Intensive Care, Charité—Universitätsmedizin Berlin, Corporate Member of Freie Universität Berlin, Humboldt-Universität zu Berlin, and Berlin Institute of Health, Charitéplatz 1, 10117 Berlin, Germany; 3 Institute of Biometry and Clinical Epidemiology, Charité—Universitätsmedizin Berlin, Corporate Member of Freie Universität Berlin, Humboldt-Universität zu Berlin, and Berlin Institute of Health, Charitéplatz 1, 10117 Berlin, Germany

**Keywords:** hepatitis C, direct-acting antivirals, kidney transplant recipients, liver stiffness, elastography, shear wave elastography, ultrasound elastography, magnetic resonance elastography

## Abstract

Hepatitis C virus (HCV) infection can lead to hepatic fibrosis. The advent of direct-acting antivirals (DAAs) has substantially improved sustained virological response (SVR) rates. In this context, kidney transplant recipients (KTRs) are of particular interest due to their higher HCV infection rates and uncertain renal excretion and bioavailability of DAAs. We investigated liver stiffness after DAA treatment in 15 HCV-infected KTRs using ultrasound shear wave elastography (SWE) in comparison with magnetic resonance elastography (MRE). KTRs were treated with DAAs (daclatasvir and sofosbuvir) for three months and underwent SWE at baseline, end of therapy (EOT), and 3 (EOT+3) and 12 months (EOT+12) after EOT. Fourteen patients achieved SVR12. Shear wave speed (SWS)—as a surrogate parameter for tissue stiffness—was substantially lower at all three post-therapeutic timepoints compared with baseline (EOT: −0.42 m/s, *p* < 0.01; CI = −0.75–−0.09, EOT+3: −0.43 m/s, *p* < 0.01; CI = −0.75–−0.11, and EOT+12: −0.52 m/s, *p* < 0.001; CI = −0.84–−0.19), suggesting liver regeneration after viral eradication and end of inflammation. Baseline SWS correlated positively with histopathological fibrosis scores (r = 0.48; CI = −0.11–0.85). Longitudinal results correlated moderately with APRI (r = 0.41; CI = 0.12–0.64) but not with FIB-4 scores (r = 0.12; CI = −0.19–0.41). Although higher on average, SWE-derived measurements correlated strongly with MRE (r = 0.64). In conclusion, SWE is suitable for non-invasive therapy monitoring in KTRs with HCV infection.

## 1. Introduction

Hepatitis C virus (HCV) primarily targets the liver, causing inflammation that can lead to long-term effects such as chronic hepatitis, cirrhosis, and hepatocellular carcinoma (HCC) [1]. Chronic HCV infections often result in liver fibrosis, which is characterized by the substitution of normal functional liver tissue with fibrous scar tissue. Stellate cell activation, signaling pathways including inflammatory cytokines, and collagen deposition in the extracellular matrix are the key factors driving this process. Despite ongoing damage, the liver’s regenerative ability slows down the progression to cirrhosis in most patients [2].

Chronic HCV infections extend beyond the liver and are associated with renal impairment, with HCV-infected patients being at a higher risk for end-stage renal disease [3]. HCV also compromises both the survival of renal transplants and their recipients [4,5]. Additionally, HCV may trigger the onset of membranous or membranoproliferative glomerulonephritis in kidney transplant recipients (KTR), leading to chronic allograft rejection [6,7].

The introduction of direct-acting antivirals (DAA) has markedly improved treatment with more than 90% of patients showing a sustained virological response (SVR) [8,9,10]. The specific DAA regimen prescribed depends on various factors, including the HCV genotype, the presence of liver cirrhosis, the patient’s treatment history, and any underlying medical condition [11]. Sofosbuvir, an NS5B inhibitor, is part of many DAA regimens. Due to its renal excretion, there were concerns about the risk of accumulation in patients with renal insufficiency. Since then, several studies have affirmed its safety and efficacy [12,13]. Liver biopsy is the reference standard for diagnosing liver fibrosis and other liver pathologies; however, it is limited by its invasive nature and associated complications, such as bleeding [14], and underdiagnosis due to sampling errors [15]. Ultrasound shear wave elastography (SWE) and magnetic resonance elastography (MRE) provide a noninvasive analysis of hepatic stiffness, which can indicate structural tissue changes in serial examinations. Several studies using MRE or SWE have already shown that hepatic stiffness is lower after DAA in HCV-infected patients [16,17,18,19,20]. High liver stiffness values (>9.2 kPa/1.75 m/s in transient elastography (TE) and >4.5 kPa/2.12 m/s in MRE) at baseline before therapy have been identified as independent predictors of HCC development [21,22].

The aim of this study was twofold: first, to evaluate the longitudinal effects of DAAs on liver stiffness in the setting of altered bioavailability in HCV-infected KTR using SWE; and second, to compare the results of SWE with the findings obtained with MRE.

## 2. Materials and Methods

### 2.1. Subjects

In this prospective, single-center study (EudraCT number: 2014-004551-32), 15 kidney transplant recipients (7 women; mean age = 48 ± 13 years) with chronic HCV infection and a clinical indication for DAA treatment were recruited at the transplant center of our hospital from December 2015 to July 2016. SWE and MRE experiments were approved by our local institutional review board (EA1/075/17), and all subjects gave written informed consent. Inclusion criteria were as follows: (i) age at least 18 years; (ii) diagnosis of chronic HCV infection of genotype Ia or Ib, defined by detectable anti-HCV antibodies and presence of an HCV RNA viral load for more than 3 months; (iii) patients not treated with or not responding to other anti-HCV treatment; (iv) estimated glomerular filtration rate (eGFR) above 30 mL/min/1.73 m^2^ for more than 12 months (estimated from blood creatinine levels using the Chronic Kidney Disease Epidemiology Collaboration (CKD-EPI) equation) [23]. Exclusion criteria were as follows: (i) contraindications to daclatasvir and sofosbuvir, co-infections such as human immunodeficiency virus or hepatitis B virus, or chronic decompensated liver disease (Child–Pugh class B or C); (ii) polycystic liver or kidney disease; (iii) history of kidney allograft rejection; (iv) history of malignancy; (v) contraindications to magnetic resonance imaging (MRI); and (vi) current participation in other drug trials, according to the study protocol of [20,24].

### 2.2. Study Protocol

**Figure 1:**
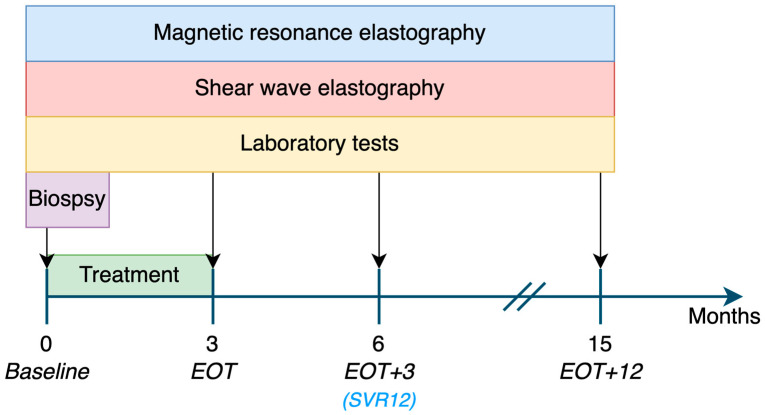
Study timeline. All participants were examined serially before treatment started (baseline), at the end of treatment (EOT), 3 months after EOT (EOT+3), and 12 months after EOT (EOT+12). Sustained virological response was achieved at EOT+3 (SVR12).

All participants were administered a daily dose of 60 mg of daclatasvir and 400 mg of sofosbuvir (direct-acting antivirals) for a duration of 3 months and clinical data were assessed according to the study protocol of the DAA safety clinical trial (Eudra-CT number: 2014–004551-32) of Duerr et al. [24]. MRE and SWE examinations were performed prior to the initiation of treatment (baseline), at the end of the treatment phase (EOT), and again at 3 (EOT+3) and 12 months after the EOT (EOT+12). Thirteen of the fifteen participants underwent a liver biopsy at the beginning of the study. However, due to the invasiveness and potential risks associated with the procedure, it was not feasible to obtain additional liver biopsies in the further course of the study; see Figure 1.

SVR was defined as an HCV RNA level ≤ 15 IU/mL. Patients who achieved SVR at 12 weeks were categorized as responders [25]. Viral relapse was defined as the reappearance of HCV with HCV RNA levels exceeding 15 IU/mL, confirmed by two consecutive positive HCV RNA analyses. In cases of viral relapse, DAA treatment was extended to 24 weeks [24]. Details are reported in [20,24].

The patients were not administered any hepatotoxic drugs and were carefully monitored for potential medication toxicities during outpatient checkups. Moreover, regular assessments of immunosuppressive drug levels were conducted to prevent toxicities. According to KDIGO guidelines [26], patients were also systematically screened for opportunistic infections (CMV, EBV).

For subsequent evaluation of MRE measurements, laboratory tests, histology, statistical data analysis, and data processing, please refer to [20]. More details on the efficacy and safety of DAA in our cohort can be found in [24].

### 2.3. Shear Wave Elastography

Acoustic radiation force impulse imaging (ARFI)-based liver SWE was used as the index test and conducted in accordance with the European Federation of Ultrasound in Medicine and Biology (EFUSMB) guidelines using a high-end ultrasound device (Aplio500, Toshiba, Otawara, Japan) with a high-frequency broadband linear transducer (5–15 MHz, centered at 10 MHz) [27]. All SWS examinations and liver biopsies were performed by the same examiner (S.R.M.G.) on day 0 (baseline). SWE was performed immediately before the ultrasound-guided biopsy to ensure a spatial match between the regions of SWE and histopathology. In brief, the patients were instructed to fast for at least two hours prior to the examination. During the examination, patients were supine with their right arm fully extended. A transducer was then positioned in the right intercostal space to visualize the right liver lobe using the B-mode technique, avoiding artifacts and large vessels during the examination. Participants were instructed to hold their breath in a neutral position and refrain from deep inspiration prior to the breath hold. The region of interest (ROI) was placed on an isoechoic region 1 to 5 cm below the liver capsule avoiding large hepatic veins and portal arteries. Using a real-time display of shear wave propagation helped in identifying appropriate regions for elasticity reconstruction. The mean SWS of each ROI was calculated, and the median of five successive measurements was then computed and expressed in m/s.

### 2.4. Magnetic Resonance Elastography

MRE was used as an imaging reference. The examinations were carried out in a 1.5T magnetic resonance imaging (MRI) scanner (Magnetom Sonata; Siemens, Erlangen, Germany). Multifrequency MRE was conducted at 10 Hz increments, spanning a range from 30 to 60 Hz. All study participants underwent consecutive SWE and MRE on the same day and were instructed to fast for at least two hours before the examinations.

Each MRE measurement was compared with the corresponding SWE measurement. Further details of the MRE technique and specific parameters are described in [20].

### 2.5. Statistical Analysis

Group mean values were calculated with their respective standard errors. Linear mixed models with random intercepts were used to account for repeated measures in the subjects. Multiple imputation using chained equations and 30 imputed datasets (imputation method: predictive mean matching, “mice v3.16.0” package) [28] was used to estimate missing values for 15 individuals. For the imputation model, we used all outcome variables and information on sex, age, and timepoint. Model-based mean differences or mean estimates for different timepoints relative to baseline, along with the 95% confidence interval (CI), are reported. All model-based estimates were adjusted for age. Correlations for repeated measures were calculated for SWS, APRI, and FIB4 using the R package “rmcorr v0.6.0” [29], with 39 degrees of freedom and 55 measures for 15 individuals. Spearman’s rank correlation coefficients were calculated for the correlation analysis between SWS and histological liver scores. Bland–Altman analysis was carried out using the R package “blandr v0.5.1”. A sample size of *n* = 14 was previously calculated for an estimated efficacy of 79% SVR12, aiming for a power of 90% with a type I error of 5% [24]. Statistical analysis was performed using SPSS Statistics for Windows, version 29 (IBM, Armonk, NY, USA), R v4.0.2/v4.3.1 (R Development Core Team), and GraphPad Prism v.9 (GraphPad software). A two-sided significance level of α = 0.05 was used. No adjustment for multiple testing was applied in this exploratory analysis; therefore, all *p*-values have to be interpreted cautiously.

## 3. Results

### 3.1. Study Population

A total of 15 kidney transplant recipients who fulfilled the inclusion and exclusion criteria were enrolled in the study; for details, see [20,24]. Fourteen of the 15 study participants achieved sustained virological response at 12 weeks (SVR12). All patients who showed a positive response to DAA treatment maintained undetectable viral RNA levels at follow-ups. Additional demographic characteristics of the study population can be found in Table 1**.** Further details of the efficacy and safety are described in [24].

Following a negative HCV-PCR at EOT, one patient experienced a viral relapse 21 days post treatment. Consequently, DAA therapy was extended to 24 weeks, resulting in an undetectable viral load at EOT+3. However, this patient encountered a second relapse 18 days after completing extended DAA treatment. Deep sequencing of the HCV genome unveiled resistance-associated variants in the NS5A regions. The patient was classified as a non-responder and an FDA-approved rescue treatment consisting of a 12-week course of sofosbuvir/velpatasvir/voxilaprevir was subsequently administered. This resulted in a decline in the viral load, with undetectable HCV-RNA persisting for 12 weeks after the completion of the rescue treatment.

Despite being classified as a non-responder, the patient was included in accordance with the methodological approach outlined in [30] for addressing outliers in datasets. Excluding this patient could introduce potential bias to the data analysis, given that the primary focus is on assessing DAA-induced changes in liver stiffness among KTRs.

### 3.2. Shear Wave Elastography

**Figure 2:**
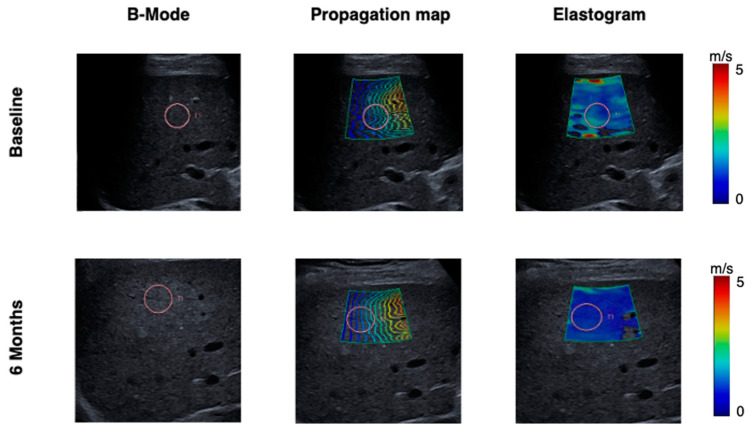
Representative ultrasound and shear wave elastography images at baseline and after 6 months (EOT+3). Shear wave speed decreased after treatment with direct-acting antivirals. The visualization of shear wave propagation in real-time allowed identification of areas with sufficient dynamic strain for precise elasticity reconstruction. The presence of a consistent and parallel wavefront pattern was considered indicative of a technically successful elastography examination.

**Figure 3:**
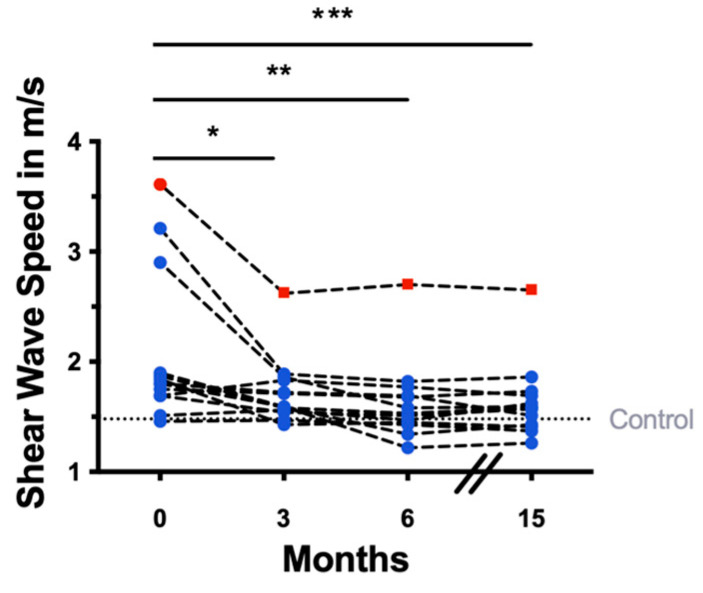
Shear wave elastography results after antiviral treatment: decrease in liver shear wave speed at 3 months (end of treatment, EOT, *n* = 13, * *p* = 0.008), 6 months (EOT+3, *n* = 14, ** *p* = 0.004) and 15 months (EOT+12, *n* = 13, *** *p* < 0.001). Red dots: persistent high SWS values in a patient who experienced viral relapses after SVR. Blue dots: patients who were categorized as responders. All *p*-values are from linear-mixed models and adjusted for age.

Substantial SWS reduction was observed both immediately after EOT and during the follow-up period. Representative B-mode images, wave propagation maps, and elastograms demonstrating the changes in liver SWS are shown in Figure 2, demonstrating the changes in liver SWS. The mean differences compared to baseline were −0.42 m/s at EOT (*p* < 0.01, CI = −0.75 to −0.09), −0.43 m/s at EOT+3 (*p* < 0.01, CI = −0.75 to −0.11) and −0.52 m/s at EOT+12 (*p* < 0.001, CI = −0.84 to −0.19). Detailed results are compiled in Table 2. Notably, the patient who experienced viral relapses consistently exhibited elevated SWS values at all four timepoints, as highlighted by red dots in Figure 3.

### 3.3. Correlation Analysis between Shear Wave Elastography, Histopathological Scores, and Serological Fibrosis Scores

**Figure 4:**
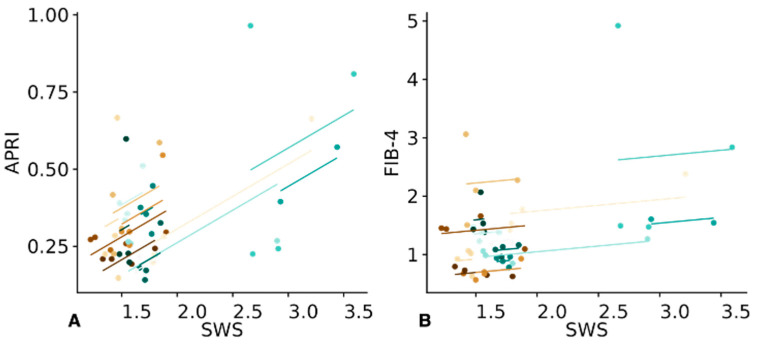
Repeated measures correlation analysis between shear wave speed (SWS) and serological fibrosis scores based on 55 measures of 15 and 39 degrees of freedom. Distinctive colors are used to represent each participant in the graph. (**A**) SWS and APRI show moderate correlation (r = 0.41, CI = 0.12 to 0.64, *p* = 0.007). (**B**) FIB-4 and SWS do not correlate substantially (r = 0.12, CI = −0.193 to 0.414, *p* = 0.448).

Liver SWS measured by SWE correlated positively with the histological fibrosis score (Spearman’s rank coefficient, r = 0.48, CI = −0.11 to 0.85, *p* = 0.05), while the (peri-)portal score did not show a considerable correlation (r = 0.33, CI = −0.31 to 0.87, *p* = 0.14). Liver SWS showed a moderate correlation with the APRI score (r = 0.41, CI = 0.12 to 0.64, *p* = 0.007) but not with the FIB-4 score (r = 0.12, CI = −0.19 to 0.41, *p* = 0.448), as shown in Figure 4.

### 3.4. Comparison between Shear Wave Elastography and Magnetic Resonance Elastography

**Figure 5:**
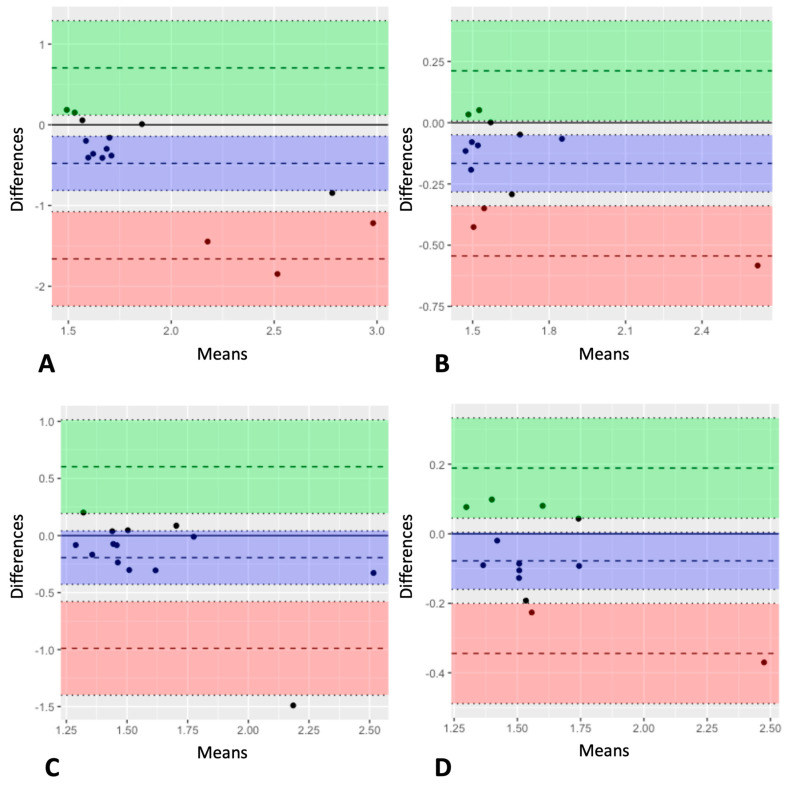
Bland–Altman plots for comparison of the two methods: shear wave speed measurements using MRE vs. SWE. *x*-axis: mean of MRI and US measurements; *y*-axis: difference between MRE and SWE (negative values indicate higher SWE-derived stiffness compared with MRE values). Horizontal lines are placed at both the mean difference and the agreement boundaries, which were calculated by adding and subtracting 1.96 times the standard deviation of the differences from the mean difference. In general, SWE yielded higher stiffness values. (**A**) Baseline, *n* = 15; (**B**) EOT, *n* = 13; (**C**) EOT+3, *n* = 14; and (**D**) EOT+12, *n* = 13.

Mostly, values obtained with SWE tended to be higher than MRE measurements, as is apparent in Bland–Altman plots for all timepoints; see Figure 5A–D. The bias and limits of agreement for the respective plots can be found in Table 3. Overall, the mean difference between SWS values was highest at baseline (−0.478; 95% CI = −0.812 to −0.144; lower limit = −1.661; upper limit = 0.705) and lowest at EOT+12 (−0.078; 95% CI = −0.160 to 0.004; lower limit = −0.344; upper limit = 0.189). Intraclass correlation coefficients (two-way mixed, absolute agreement) results also varied similarly between the different timepoints. At baseline (*n* = 15), the ICC was 0.33 (95% CI: −0.10 to 0.69) and improved to 0.74 (95% CI: 0.17 to 0.92) at EOT (*n* = 13). Although the ICC slightly decreased to 0.45 (95% CI: 0.02 to 0.78) at EOT+3 (*n* = 14), it markedly increased to 0.88 (95% CI: 0.62 to 0.96) at EOT+12 (*n* = 13). More details regarding MRE results are provided in [20].

**Figure 6:**
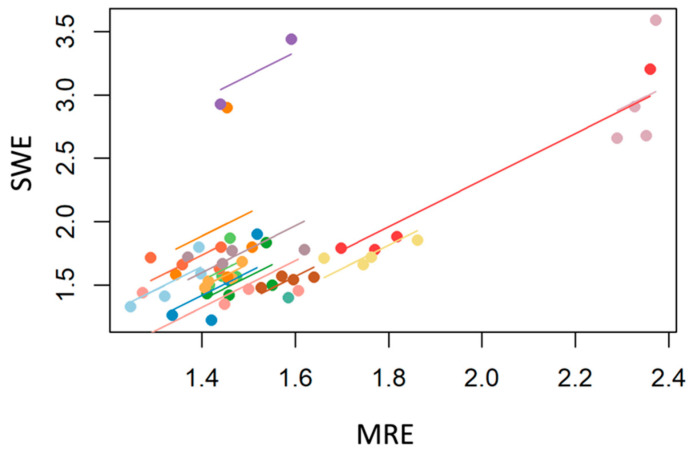
Repeated measures correlation analysis shows a substantial and positive correlation between SWE and MRE (r = 0.64; CI = 0.41 to 0.80) based on 55 measures of 15 individuals and 35 degrees of freedom. Distinctive colors are used to represent each participant in the graph.

As shown in Figure 6, analysis of repeated measures correlation with 35 degrees of freedom and 55 measures of 15 individuals revealed a positive correlation between SWE and MRE (r = 0.64; 95% CI = 0.41 to 0.80).

## 4. Discussion

This study aimed to investigate the effects of DAAs on liver stiffness in renal transplant patients with HCV infection. We measured changes in liver stiffness using SWE and compared the results with those obtained by MRE [20].

Overall, our findings demonstrate a decrease in liver stiffness after DAA therapy in KTRs with HCV. This reduction in liver stiffness suggests that DAA treatment is effective in reducing liver fibrosis and inflammation. Notably, the patient who experienced a relapse, after an initial negative RNA-PCR at EOT, showed elevated SWS values throughout the follow-up period, and was detected both by SWE and MRE [20]. This observation is similar to the findings of Goertz et al. [31], who also reported relapses in patients with consistent SWS values.

Further studies are needed to determine the predictive significance of constantly high SWS values in anticipating future relapses or treatment failure.

MRE is considered the noninvasive in vivo reference standard for hepatic viscoelasticity as it analyzes shear waves throughout the entire liver [19]. It is mostly operator-independent and suitable for assessing unevenly distributed hepatic pathology [32]. SWE, when integrated into state-of-the-art ultrasound devices, is economical, quick, and simple, making it accessible for routine clinical use [33].

Interestingly, while both methods detected substantial decreases in SWS values at EOT+3 and EOT+12, only SWE identified a substantial decrease at EOT as well. Moreover, SWE frequently yielded higher SWS values than MRE, which might be explained by the viscoelastic properties of liver tissue. As shown in [34], viscoelastic dispersion results in higher stiffness values when the shear wave’s center frequency is above 200 Hz (as in SWE) compared to below 60 Hz (in MRE). Bland–Altman and ICC analyses showed similar patterns, with the highest agreement observed at EOT+12 and the lowest at baseline, where the inflammation-induced dispersion in the latter might lead to even higher variance. Nonetheless, repeated measures correlation analysis revealed a positive correlation between SWE and MRE measurements (r = 0.64), emphasizing that SWE and MRE allow consistent assessment of liver stiffness in KTRs. A prior retrospective study further confirmed a high degree of clinical interchangeability between 2D-SWE and MRE, reporting an ICC of 0.82 [35]. However, when comparing SWE and MRE to the histopathological fibrosis score—regarded as the gold standard for assessing liver fibrosis [36]—SWE showed a stronger correlation. The Spearman correlation coefficients between SWE and the histopathological fibrosis score (r = 0.48) exceeded those previously reported between MRE and the latter (r = 0.36) [20].

We reported shear wave speed in m/s without conversion to Young’s modulus in kPa to avoid potential inaccuracies in the conversion process. To facilitate comparisons with other studies, we converted kPa back to m/s using the formula E = 3ρc, where ‘c’ is shear wave speed, and ‘ρ’ is tissue density standardized at 1 kg/m^3^ [27]. Previous studies have reported baseline values ranging from 1.28 to 2.46 m/s for ARFI [18,37,38,39,40,41], 1.45 to 3.29 m/s for TE [12,17,18,38,42,43,44,45,46,47,48,49], and 1.76 to 2.05 m/s for MRE [50,51,52]. Our baseline values also fall within this reported range. Differences between baseline and EOT in previous studies ranged from −0.12 to −0.20 m/s for ARFI [18,39,40], −0.02 to −0.63 m/s for TE [17,18,45,46,47], and −0.23 m/s for MRE [51]. Differences between baseline and EOT+3 were −0.60 m/s for ARFI [18], −0.24 m/s for TE [18], and −0.11 m/s for MRE [52]. Lastly, the described differences between baseline and EOT+12 ranged from −0.42 to −0.50 m/s for ARFI [18,37] and −0.31 to −0.32 m/s for TE [18,46]. The decrease in stiffness observed in our study matched the reported ARFI-based differences for both EOT+3 and EOT+12 compared to baseline. These variations in stiffness values between studies might be attributable to differences in fibrosis grades. Patients with higher fibrosis grades tend to exhibit a more prominent reduction in stiffness [40,53]. The early post-therapeutic reduction in liver stiffness might be attributable to the resolution of necroinflammation [47,54]. In contrast, fibrosis tends to improve slowly at a later stage [41]. It is also worth noting that different optimal cut-off values for fibrosis detection have been proposed depending on the specific elastography device in use [27]. Nonetheless, measurements from TE were shown to correlate strongly with those from five different ARFI-based ultrasound devices, with Pearson’s correlation coefficients ranging from 0.86 to 0.95 [55].

Several DAA-based regimens are currently available, with nearly all combinations of DAAs demonstrating high sustained virologic response (SVR12) rates and low rates of adverse events in chronic kidney disease patients, those undergoing dialysis, and KTRs. Sofosbuvir, which is still the backbone of many DAA regimens in developing countries, was also found to be safe. KTRs with eGFR > 30 mL/min/1.73 m^2^, similar to our study cohort, experienced both high efficacy and safety with the evaluated regimens, including daclatasvir/sofosbuvir [56]. The DAA regimen we employed is particularly advantageous in KTRs due to minimal drug interactions, as evidenced by studies showing that daclatasvir, despite being a CYP3A4 substrate, and sofosbuvir do not impact the pharmacokinetic parameters of critical immunosuppressive medications such as tacrolimus, everolimus, or cyclosporine [57,58].

This study has several limitations. A larger sample size would yield more reliable evidence regarding the effects of DAA therapy on liver stiffness and support the exploration of the trends observed in our exploratory analysis. Furthermore, a longer follow-up duration is needed to confirm the long-term persistence of the observed changes in our measurements, since ours was limited to one year post-therapy. Additionally, due to their invasive nature, liver biopsies were only obtained at the beginning of the study. Instead, surrogate parameters were regularly measured, consistently demonstrating a positive correlation between hepatic SWS and fibrosis [20].

In conclusion, our study shows a sustained reduction in liver stiffness over one year in HCV-infected KTRs following DAA therapy, with a strong correlation between SWE and MRE. These results suggest effective viral suppression and the potential for affordable long-term ultrasound-based monitoring of KTRs.

## Figures and Tables

**Table 1:** Demographic characteristics and selected HCV-related data of the study population.

Characteristics	Kidney Transplant Recipients
Number of participants	15
Number of men	8
Number of women	7
Age in years Mean (SD)	48 (13)
Body mass index (kg/m^2^) Mean (SD)	23.3 (4.5)
Time since kidney transplantation in years Mean (SD)	13.1 (6.9)
HCV RNA level at baseline (10^6^ × IU/mL) Mean (SD)	1.73 (1.28)
Treatment-to-clearance interval (days) Median (IQR)	20 (11–28)

SD = standard deviation. IQR = interquartile range. According to [20].

**Table 2:** Shear wave elastography.

	Baseline (*n* = 15)	EOT (*n* = 13)	EOT+3 (*n* = 14)	EOT+12 (*n* = 13)
Shear wave speed (m/s) Mean (SD) Difference compared to baseline Mean (95% CI)	2.14 (0.74)	1.73 (0.38) * −0.42 (−0.75 to −0.09)	1.71 (0.49) ** −0.43 (−0.75 to −0.11)	1.63 (0.34) *** −0.52 (−0.84 to −0.19)

SD = standard deviation; CI = confidence interval; EOT = end of treatment; EOT+3 = 3 months after end of treatment; EOT+12 = 12 months after end of treatment. Compared to baseline: * *p* = 0.008, ** *p* = 0.004 and *** *p* < 0.001. All *p*-values are from linear-mixed models and adjusted for age.

**Table 3:** Results of Bland–Altman analysis.

	Baseline (*n* = 15)	EOT (*n* = 13)	EOT+3 (*n* = 14)	EOT+12 (*n* = 13)
Bias (95% CI)	−0.478 (−0.812 to −0.144)	−0.166 (−0.283 to −0.050)	−0.193 (−0.428 to 0.041)	−0.078 (−0.160 to 0.004)
ULoA (95% CI)	0.705 (0.121 to 1.289)	0.212 (0.007 to 0.416)	0.602 (0.192 to 1.013)	0.189 (0.045 to 0.333)
LLoA (95% CI)	−1.661 (−2.245 to −1.076)	−0.544 (−0.749 to −0.340)	−0.989 (−1.400 to −0.578)	−0.344 (−0.489 to −0.200)

ULoA = upper limit of agreement; LLoA = lower limit of agreement; 95% CI = 95% confidence interval; EOT = end of treatment; EOT+3 = 3 months after end of treatment; EOT+12 = 12 months after end of treatment. The limits of agreement were calculated by adding and subtracting 1.96 times the standard deviation of the differences from the mean difference.

## Data Availability

The data presented in this study are available on request from the corresponding author. The data are not publicly available due to data privacy.
